# RNA Fluorescence with Light-Up Aptamers

**DOI:** 10.3389/fchem.2016.00029

**Published:** 2016-06-28

**Authors:** Jonathan Ouellet

**Affiliations:** Department of Chemistry and Physics, Monmouth UniversityWest Long Branch, NJ, USA

**Keywords:** light-up aptamers, Spinach, Broccoli, Mango, RNA fluorescence, green RNA

## Abstract

Seeing is not only believing; it also includes understanding. Cellular imaging with GFP in live cells has been transformative in many research fields. Modulation of cellular regulation is tightly regulated and innovative imaging technologies contribute to further understand cellular signaling and physiology. New types of genetically encoded biosensors have been developed over the last decade. They are RNA aptamers that bind with their cognate fluorogen ligands and activate their fluorescence. The emergence and the evolution of these RNA aptamers as well as their conversion into a wide spectrum of applications are examined in a global way.

## Introduction

Cellular imaging techniques have been essential to understand spatial and temporal resolution in molecular and cellular biology. Green fluorescent protein (GFP) and its derivatives have been central to these efforts by tagging onto endogenously coding genes of proteins to map the expression, interaction, localization, and role of proteins in the cell and beyond (Enterina et al., 2015; Mishin et al., 2015). Historically, proteomics and protein-centered research has been emphasized for many years and there has been little need to move away from GFP technologies. We are in a genomic revolution and our understanding of the role of RNA in the cell and beyond are expanding. The vast amount of innovation in the RNA field is reshaping prior conceptions about RNA, and new tools are being developed as the field evolves. An example would be the MS2-GFP system developed around the year 2000 (Bertrand et al., 1998; Fusco et al., 2003), in which imaging of tagged endogenous RNA in living cells was performed using 24 units of MS2 aptamers (repeated stem-loops that are specifically bound by a coat protein of the bacteriophage MS2), which therefore accumulate MS2-GFP fusion proteins on the mRNA to turn it fluorescent. However, the background fluorescence from unbound MS2-GFP greatly affects the signal-to-noise ratio. A “traditional” method of RNA imaging has been fluorescent *in situ* hybridization (FISH), which requires short exogenous fluorescent oligonucleotide probes complementary to a targeted cellular RNA sequence. However, fixing the cells before hybridization followed by extensive washes of the unbound probes prevent monitoring the critical dynamic regulation aspects of RNA. Such steps were circumvented with the development of molecular beacons where a quencher and a fluorophore, positioned at both 5′-and 3′-ends RNA extremities of a stem-loop hairpin structure, would become fluorescent once bound to its cognate complementary RNA strand (Santangelo, 2010). Exogenously added probes also had their share of drawbacks; the use of endogenously produced RNA would provide greater advantages.

More than a decade ago, an RNA aptamer was uncovered using systematic evolution of ligands by exponential enrichment (SELEX) to enhance the fluorescence of a compound otherwise practically non-fluorescent (Grate and Wilson, 1999). Unfortunately, the cytotoxicity of malachite green (MG) hindered the development of this aptamer for *in vivo* imaging (Kraus et al., 2008). It however was the first endogenously produced RNA inducing fluorescence upon binding of an otherwise non-fluorescent small molecule. In recent years, the light-up aptamers field rapidly progressed and its strengths and weaknesses are now assessed.

**Figure 1:**
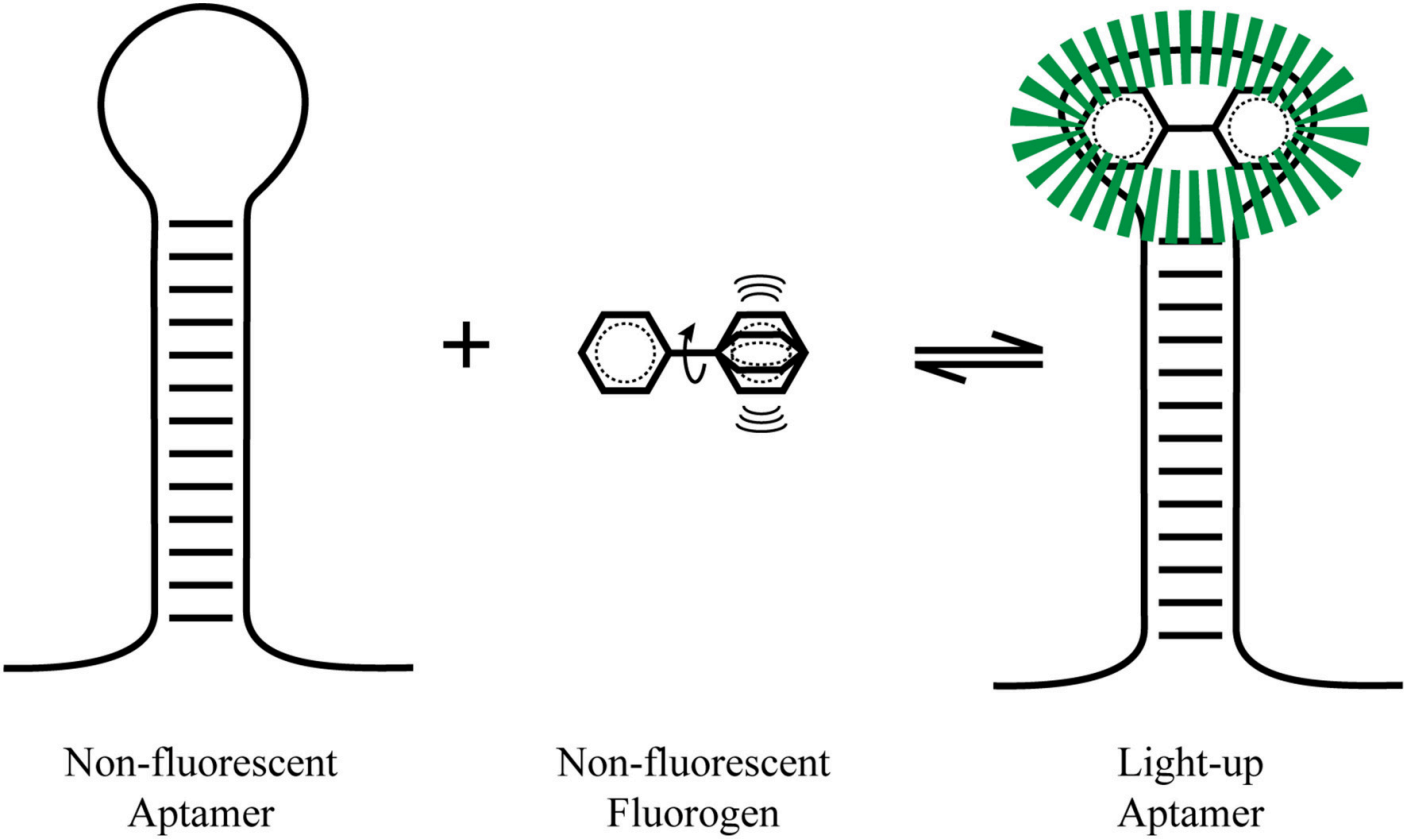
**Light-up aptamers**. A non-hindered fluorogen can be excited and have its energy dissipated by non-radiative pathway such as molecular vibrations (heat). Once tightly bound by an aptamer, the fluorophore is stabilized and radiative fluorescence decay pathways predominate, leading to a large fluorescence increase.

A major advantage of light-up aptamers over other imaging techniques (such as FISH, GFP, molecular beacons) is the low fluorescent background (leading to a high signal-to-noise ratio) in the absence of the RNA aptamer. The strong resurgence of the aptamer generation and selection field brought new tools to synthesize novel type of aptamers that would improve the area of *in vitro* and *in vivo* RNA imaging. For these light-up aptamers, the role of the aptamer appears to be the stabilization of the planar structure of the small molecule to prevent the dissipation of its energy through non-radiative decay pathways (such as heat, thru molecular motions), so that radiative decay pathways (such as fluorescence, phosphorescence) can predominate, leading to an increase in fluorescence (Figure 1).

These light-up aptamers allow the tagging of RNAs, thus providing a simple approach for a simple protein-free live-cell imaging of RNAs by fluorescence. Being composed of RNA, light-up aptamers have a huge advantage over GFP. RNA is highly modular (Kertsburg and Soukup, 2002) and its sequence can be rapidly optimized by cycles of transcriptions, selection, and reverse transcription (Ellington and Szostak, 1990; Robertson and Joyce, 1990; Tuerk and Gold, 1990). Since RNA aptamers can be generated against virtually any target molecule, development of allosteric light-up aptamers can be a genuine alternative to primary and secondary antibody chemistry. Moreover, traditional studies of the modulation of gene expression often rely on quantifying GFP, which must be transcribed and translated, delaying detection by 10-30 min after initial gene transcription. Light-up aptamers provide a direct measure of gene transcription at the RNA level that reveals a temporally more accurate observation of RNA localization and real time promoter activity.

This review offers a current update of the light-up aptamer progress and applications such as RNA trafficking, metabolite sensing via a transducer linked to metabolite-sensing aptamers, protein sensing, RNA, and ribonucleoprotein (RNP) purification, targeted RNA detection via strand displacement, ribozyme cleavage monitoring, and development of demethylase inhibitors.

## The beginnings of light-up aptamers

A malachite green (MG) aptamer was developed to confine the formation of radicals on RNA upon laser activation, leading to the rapid degradation of such RNA (Grate and Wilson, 1999). Other research groups studied this new type of aptamer where the fluorescence intensity of MG increased when bound to the malachite green aptamer (MGA). One in particular had the long-term aim to use aptamers to bind fluorogens (small non-fluorescent ligands that conditionally become fluorescent) to activate their fluorescence as a new protein-free tool to monitor RNAs *in situ* (Babendure et al., 2003). They monitored the fluorescence activation of MG and other triphenylmethane derivatives upon binding by the 38–54 nucleotides aptamer. A 2300 fold fluorescence increase upon RNA binding lead to fluorescence in the GFP brightness range (Table 1). A crystal structure (RCSB Protein Data Bank, accession identifier 1F1T) of the 38 nucleotide aptamer bound to TMR (tetramethylrhodamine) suggests a stacking of the TMR onto a base quadruplex; stabilizing its intramolecular motions (Baugh et al., 2000). However, the intrinsic property of MG to form radicals upon excitation makes it susceptible to cytotoxicity. The effects of MG (and several analogs) on cell growth and apoptosis in yeast and human ovarian cells have been explored, where the limiting toxic concentration is around 0.1 to 1 μM (Kraus et al., 2008). Although the malachite green cytotoxicity effects has prevented detailed *in vivo* imaging, this type of fluorescence activation has opened this field of research, pulling in several research groups to refine the tool.

**Table 1:** **Photophysical and binding properties of fluorophore-aptamer complexes**.

**Fluorogen**	**Aptamer**	**λex (nm)**	**λem (nm)**	**ε^a^ (M^−1^cm^−1^)**	**φ^b^**	***K*^c^_D_ (nM)**	**Brightness^d^**	**References**
Aeguorea GFP	–	395	508	27600	0.79	–	21800	Paige et al., 2011
EGFP	–	489	508	55000	0.60	–	33000	Paige et al., 2011
Malachite green	–	618			7.9 × 10^−5^	–		Babendure et al., 2003
	MGA	630	650	150000	0.19	117	28500	Babendure et al., 2003
Hoechst 1C	–	345	470	N.D.	0.017	–		Sando et al., 2008
	BFR	345	470	N.D.	0.26	35	N.D.	Sando et al., 2008
DIR	–			134000		–		Constantin et al., 2008
	DIT-Apt1	600	~650	N.D.		86	N.D.	Constantin et al., 2008
DMHBI	–	394	487	23336	0.0005	–	12	Paige et al., 2011
	2–4	397	464	21536	0.10	N.D.	21500	Paige et al., 2011
	13–2	398	529	23391	0.06	464	1400	Paige et al., 2011
	13–2-min	398	529	23391	0.11	N.D.	2600	Paige et al., 2011
	3–6	398	537	25300	0.05	406	1300	Paige et al., 2011
	17–3	405	547	18127	0.09	N.D.	1600	Paige et al., 2011
DMABI	–	447	519	14329	0.003	–	40	Paige et al., 2011
	11-3	489	512	18743	0.05	N.D.	900	Paige et al., 2011
2-HBI	–	399	461	10943	0.0005	–	5	Paige et al., 2011
	6–8	396	588	10714	0.004	N.D.	43	Paige et al., 2011
DFHBI	–	405	498	11864	0.0007	–	8	Paige et al., 2011
	Spinach (24–2)	452	496	24271	0.72	537	17000	Paige et al., 2011
	Spinach2	454	498	26100	0.70	430	18000	Strack et al., 2013
TO1-Biotin	–	500	525	N.D.	0.0002	–	N.D.	Dolgosheina et al., 2014
	Mango	510	535	77500	0.14	3.2	11000	Dolgosheina et al., 2014
TO3-Biotin	–							Dolgosheina et al., 2014
	Mango	637	658	N.D.	N.D.	6–8		Dolgosheina et al., 2014
DFHBI-1T	–	426	495	35400	0.00098		35	Filonov et al., 2014
	Spinach2	482	505	31000	0.94	560	29000	Song et al., 2014
	Broccoli	472	507	29600	0.94	360	27800	Filonov et al., 2014
DFHBI-2T	–	460	515	34800	0.0012		50	Song et al., 2014
	Spinach2	500	523	29000	0.12	1300	3000	Song et al., 2014
PFP-DFHBI	–							Ilgu et al., 2015
	Spinach2					160		Ilgu et al., 2015

a *Molar extinction coefficient*.

b *Fluorescence quantum yield*.

c *Dissociation constant for the fluorophore-aptamer complex*.

d *Brightness is the product between the molar extinction coefficient and the fluorescence quantum yield*.

*N.D., not determined*.

In 2008, several new light-up aptamers were developed. Derivatives of thiazole orange (TO) and dimethyl indole red (DIR) were synthetized and showed little nonspecific fluorescence activation from cell lysate (Constantin et al., 2008). A 102-nucleotide aptamer (DIR-Apt1) was developed using SELEX and showed a 60 fold fluorescent enhancement of the new cyanine dye. Soon after, a derivative of Hoechst, Hoechst-1C [*N*-(6-aminohexyl)-2-(2,6-di-*tert*-butyl-4-(5-(4-methylpiperazin-1-yl)- 1*H*,1′*H*-2,5′-bibenzo[*d*]imidazol-2′-yl)phenoxy)acetamide] fluoresced only in the presence of a 71 nucleotide-long aptamer which were minimized and optimized to a 29 nucleotide-long RNA blue fluorescent aptamer (BFR; Sando et al., 2008). Importantly, it was successfully used to monitor *in vitro* transcription. Considering the inherent nature of the Hoechst dye to bind DNA, its binding specificity was still unclear with respect to whether the derivative would prove non-toxic and strictly aptamer-specific in a cellular context.

## The breakthrough

**Figure 2:**
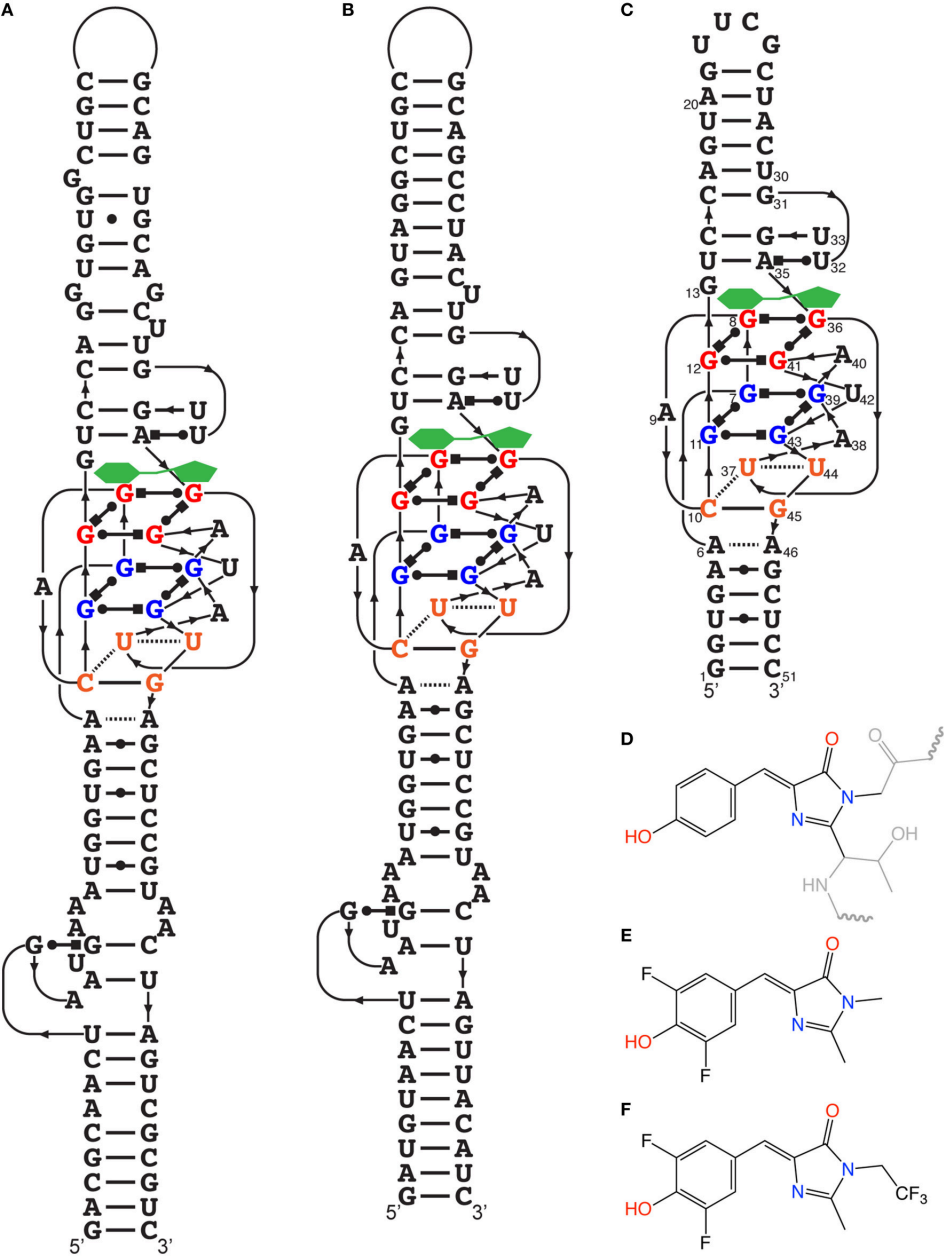
**Spinach and HBI and their optimized derivatives**. **(A)** Secondary structure of the RNA aptamer Spinach determined from its crystal structure. The fluorogen (green) is stabilized on top of the G-quadruplex structure. **(B)** Secondary structure temperature-optimized Spinach2. **(C)** Deduced secondary structure of Baby Spinach engineered from the crystal structure essential features. **(D)** Structure of HBI stabilized within GFP, where the gray regions represent the amino acid segments not involved in the fluorescence. **(E)** Structure of DFHBI, the fluorogens used for most studies in this review. **(F)** Structure of DFHBI-1T, the fluorogens with spectral properties more adapted to optics used with GFP fluorescence.

Within the laboratory of S.R. Jaffrey, attention was given to the components of GFP that make it fluoresce. Several elements of the photochemistry of GFP are known, such that the amino acids Ser65-Tyr66-Gly67 undergo autocatalytic intramolecular cyclization, resulting in 4-hydroxybenzylidene imidazolinone (HBI; Figure 2D). Free HBI or a denatured GFP do not produce fluorescence. The structure of the GFP protein is used as a scaffold to form specific tertiary interaction to stabilize HBI, making fluorescence the major pathway to dissipate the energy from the excited state of the fluorophore (Conyard et al., 2011). Therefore, they synthetized several HBI derivatives (fluorogens resembling the one found in GFP) having very low fluorescence in cells, and identified RNA aptamers by performing column-based SELEX (Paige et al., 2011). In doing so, they found several aptamers binding to the HBI derivatives, covering a large color spectrum (Table 1). Among others, 3,5-difluoro-4-hydroxybenzylidene imidazolinone (DFHBI) showed a remarkably enhanced green fluorescence when in presence of a 98 nucleotide-long aptamer and was given the name of the green vegetable, Spinach (Figures 2A,E). To verify the feasibility of in vivo imaging, the comparison of the fluorogens DFHBI, TO, MG and 4-dimethylamino-4′-nitrostilbene (DANS) were incubated in cells and tested for their nonspecific cellular fluorescence. Except for DFHBI they all exhibited nonspecific fluorescence in cells, and importantly, DFHBI did not induce cytotoxicity or phototoxicity. This was a critical step to move toward in vivo imaging using RNA aptamer technology. Additionally, an example of its application was studied. The 5S rRNA was tagged with Spinach and its trafficking in living mammalian cells was observed by fluorescence microscopy, thus confirming the membrane permeability of DFHBI (Paige et al., 2011). While a GPF-tagged protein typically requires 10–100 ms of exposure time, the highly expressed 5S-Spinach required 1 s. This increase in exposure time is related to thermal instability and poor folding of this RNA aptamer in vivo. Although the permeability, non-toxicity, and fluorescence intensity increase were major advances in the RNA aptamer fluorophore design and technology, the thermal instability of the RNA aptamer required improvement.

## Optimization of spinach

**Figure 3:**
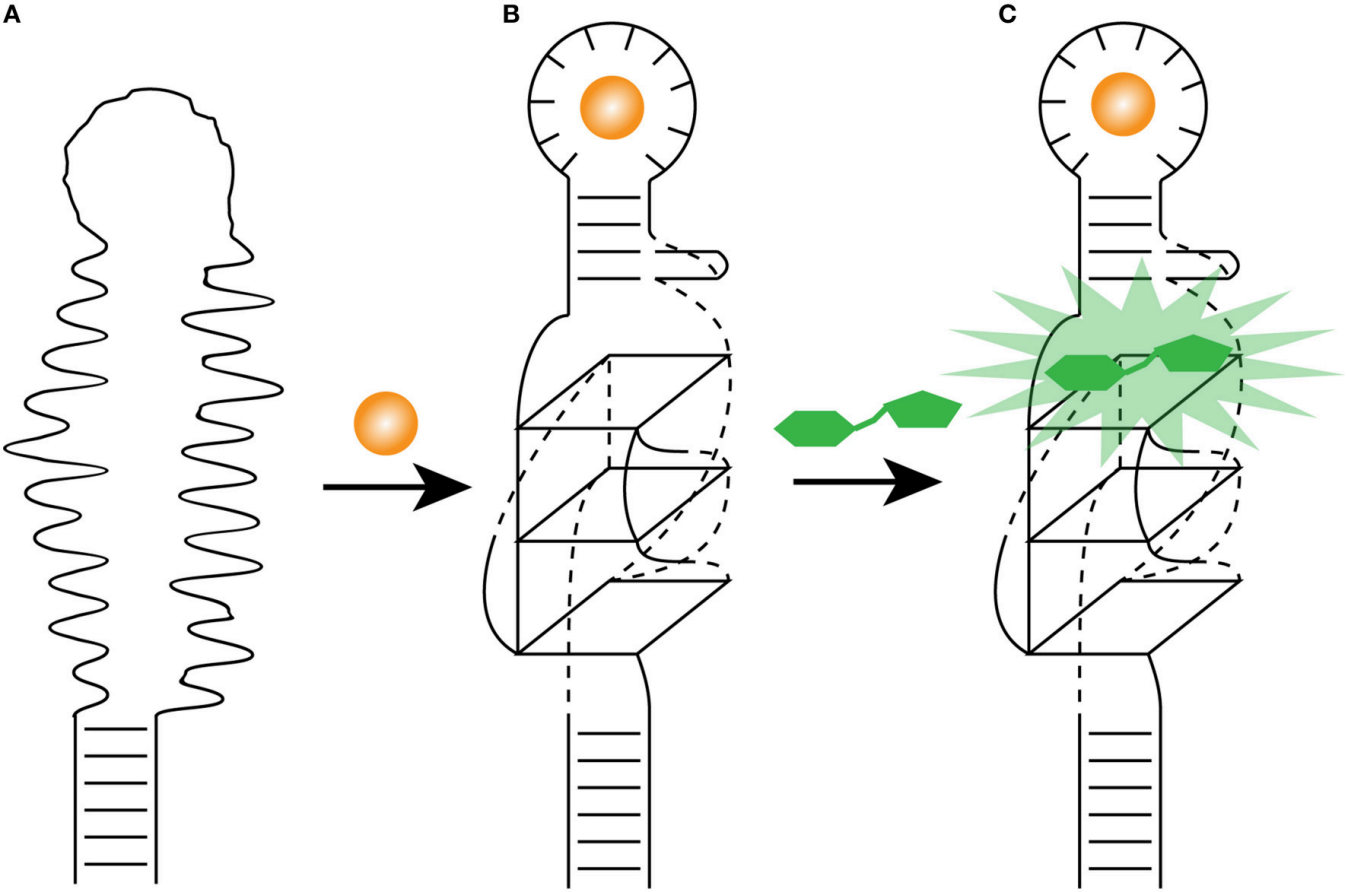
**Light-up aptamer as biosensors. (A)** The engineered allosteric light-up aptamer is in a misfolded state and cannot bind a fluorogens. **(B)** Once the metabolite (orange sphere) binds the metabolite-binding aptamer, it promotes the folding of its own stem, which in turns stabilize the formation of the G-quadruplex. **(C)** The presence of the essential G-quadruplex and its surrounding nucleotides allows the fluorogen (green) to bind the modified light-up aptamer and releases its energy as fluorescence.

Functional RNAs are highly modular (Kertsburg and Soukup, 2002), where independently folded domain (harboring a specific activity) can be relatively easily fused to other RNA molecules with distinct function, creating an RNA with dual activity. This type of structural modification of Spinach has changed the field of protein-free *in vivo* metabolite sensing. The previous minimization experiments of Spinach (to 71 nucleotides) indicated that a stem-loop can be shortened (Paige et al., 2011). In order to transform Spinach as a metabolite-sensing tool, the stem was replaced by a selected transducer stem thereby linking Spinach to an aptamer that is known to bind a specific metabolite (Paige et al., 2012). Without the metabolite, the transducer stem is not formed, leading to a Spinach molecule that cannot bind DFHBI. Upon metabolite binding, the transducer stem forms and stabilizes Spinach that can bind DFHBI, resulting in a fluorescence signal (Figure 3).

Using the S-adenosylmethionine (SAM) aptamer connected to Spinach *via* a transducer, the dynamics of SAM production were monitored *in vivo*. The sensor exhibited minimal fluorescence in cells deprived of methionine while a 6-fold fluorescence increase was observed when methionine was provided. Similarly, sensors were developed to observe the dynamics in live *E. coli* of adenosine 5′-diphosphate (ADP), guanine, and guanosine triphosphate (GTP). Under certain conditions, these sensors produced ~20-fold increases in fluorescence upon metabolite binding. Moreover, SAM synthesis that was monitored in individual *E. coli* cells was highly variable from cell-to-cell, once again pointing the importance of population averaging *versus* single cell measurements.

A low melting temperature (*T*_*m*_) of 34°C was central in the poor folding of Spinach where an estimated 32% of the aptamer was folded at 25°C while only 13% was at 37°C. To increase Spinach thermostability and brightness, systematic mutagenesis was performed on the aptamer, resulting in a more stable light-up aptamer appropriately named Spinach2 (Figure 2B; Strack et al., 2013). Moreover, previous studies indicated that the brightness was drastically reduced from the *in vitro* to *in vivo* transition. The fusion of Spinach or Spinach2 to the ${\text{tRNA}}_{3}^{\text{Lys}}$ sequence greatly increase the fraction of folded aptamers as the flanking sequence acts as a folding scaffold (Ponchon and Dardel, 2007). The fluorescence of tagged RNAs with Spinach was successfully monitored in HeLa cells as well as in HEK293T cells showing diffuse nuclear, cytoplasmic distribution or speckles depending on the identity of the tagged RNA. Time-lapse imaging revealed that foci were mobile and can be merged to form larger foci. Perhaps more interesting in terms of dynamics, it was observed that tautomycin (an inhibitor of protein phosphatases PP1 and PP2A as well as an immunosuppressor) induces disaggregation of foci in as little as 1 h. Most of the research on the optimization of the Spinach aptamer and its ligand were done in parallel and therefore, some of the progress mentioned were done with the “original” Spinach rather than Spinach2. The two systems being very closely related, most of the improvements can be transferred from Spinach to Spinach2.

A logical way to improve the brightness of Spinach is to better understand its photochemistry. In state-of-the-art single-molecule experiments (requiring powerful excitation), where the excitation of a single spinach RNA attached to a slide in presence of 5 μM of DFHBI, the bright fluorescence of the Spinach-DFHBI complex showed fast decay (to lower than 5% of the initial level) after only 2 s of excitation at ~100 W/cm^2^. However, a pulsed excitation showed that about 90% of the fluorescence was recovered when performed on a slide as well as in *E. coli*, while using the same excitation intensity (Han et al., 2013). This ruled-out irreversible photobleaching of the fluorophore but rather suggested a different photophysical model. It was proposed that upon illumination, the bound DFHBI undergoes photoisomerization, leading to a fast unbinding from the aptamer; followed by the binding of a new DFHBI. Pulsed illumination at 0.2 Hz (a 50 ms illuminations followed by 5 s dark) allows sufficient recovery time for the complex, where the fluorescence signal does not decrease significantly over 150 s, whereas the fluorescence signal disappear after 0.2 s under similar conditions with a continuous-wave illumination. Although light-up aptamer applications in cells are not routinely performed at the single RNA level (where the high laser excitation intensity dramatically increase the fluorescence decay), it should be noted that the cumulative fluorescence intensity from the ensemble comes from all the individual single events. The optimization of single-events from pulsed illumination leads to brighter aptamer over a longer period of time. Pulse illumination should be therefore considered for *in vivo* imaging where most applications require long exposition to measure slow cellular events.

In terms of microscopy, a drawback of the Spinach-DFHBI fluorescence is that its spectral properties are different from current optical instrumentations that are optimized for GFP or fluorescein isothiocyanate (FITC) with a bandpass excitation filter transmitting light at 480 nm, a 505 nm dichroic mirror and an emission filter transmitting light at 535 nm. With a maximal emission wavelength slightly below 500 for the Spinach-DFHBI complex, most of the fluorescent light is not collected by the dichroic mirror or is blocked by the emission filter. During the initial development of HBI derivatives (Paige et al., 2011), it was clear that functional groups modifying the delocalization of the electrons changed the spectral properties of the fluorescence spectra. Addition of a trifluoroethyl substituent on a methyl group (DFHBI-1T; Figure 2F) or on carbon 2 (DFHBI-2T) allowed an increase of brightness for Spinach2-DFHBI-1T (but with similar optical properties than DFHBI), while Spinach2-DFHBI-2T offered ideal optical properties for YFP filters (rather than GFP), but with a poor fluorescent quantum yield, leading to a 5-fold decrease in brightness (Song et al., 2014). If one has access to a microscope with modular optics, DFHBI-1T should now be the fluorogens of choice while a fixed standard YFP filter system would collect more light with DFHBI-2T albeit the loss of the fluorescence quantum yield.

As it is the case for many functional RNAs, the elucidation of their crystal structures is often fundamental to understanding their inner workings. The crystal structure of Spinach revealed that bound DFHBI is stabilized among a base triple, a G-quadruplex and an unpaired G (Warner et al., 2014), rather than the initially proposed four-way junction (RCSB Protein Data Bank, accession identifier 4TS0 and 4TS2). It is a G-quadruplex of unique topology as it is connected on both sides by antiparallel A-form duplexes. One of these stems was the one in which a sensing aptamer was fused (Paige et al., 2012). Moreover, the determination of the Spinach structure allowed a rational reduction in the size of the non-conserved stems to a minimal Spinach of 51 nucleotides appropriately called Baby Spinach (Figure 2C). The presence of a G-quadruplex is ideal to create a large planar platform by which the planar fluorophore can be stabilized *via* multiple weak interactions.

The stability of engineered RNAs *in vivo* is a major issue and the initial Spinach experiments (Paige et al., 2012) employed a tRNA scaffold to promote folding as well as to enhance the stability of the Spinach aptamer (Ponchon and Dardel, 2007). Although the tRNA scaffold provided a stronger *in vivo* fluorescence signal, a deeper analysis indicated RNA degradation. Using Spinach for in-gel imaging (see in section below for more details on the application), the ${\text{tRNA}}_{3}^{\text{Lys}}$ scaffold induced a 3′ cleavage in bacteria, while it is targeted by endonucleases in mammalian cells (Filonov et al., 2015). This susceptibility to endonuclease was also confirmed by a shorter 5S RNA half-life when it was in presence of the tRNA scaffold. To test whether the identity of the tRNA scaffold would influence the stability and degradation, two other scaffolds were used for their known scaffolding capacity and stability of previously engineered RNA, namely V5 from *Vibrio proteolyticus* 5S rRNA scaffold (Zhang et al., 2009) and F29 RNA three-way junction motif (Shu et al., 2014). V5-Broccoli showed cleavage while F29-Broccoli was mostly a single transcript in *E. coli*, while the opposite occurred in mammalian cells. A putative transcription termination site in F29 was eliminated by engineering a double mutation, resulting in the RNA Scaffold F30. Analysis of F30-Broccoli expression in mammalian cells and in bacteria showed no or minimal degradation (Filonov et al., 2015).

The several modifications (discussed above) integrated into Spinach to improve the brightness were still questionable for the detection of mRNA expressed at low-level (transcribed by the RNA polymerase II). To improve on the fluorescence signal, an array of Spinach aptamer repeats was created. Arrays of up to 64 Spinach repeats increased by 17-fold the fluorescence signal which allowed for imaging mRNAs that would otherwise be invisible with a single aptamer tag (Zhang et al., 2015). An array of 32 Spinach repeats yields a fluorescence intensity comparable to that of RFP (red fluorescence protein) where both mRNA where expressed at the same level as measured by qPCR. Considering the shorter sizes and higher efficiencies of Spinach2 and other novel aptamers (see below), it would be interesting to see them arranged in such an array as well.

## Development of novel light-up aptamers

**Figure 4:**
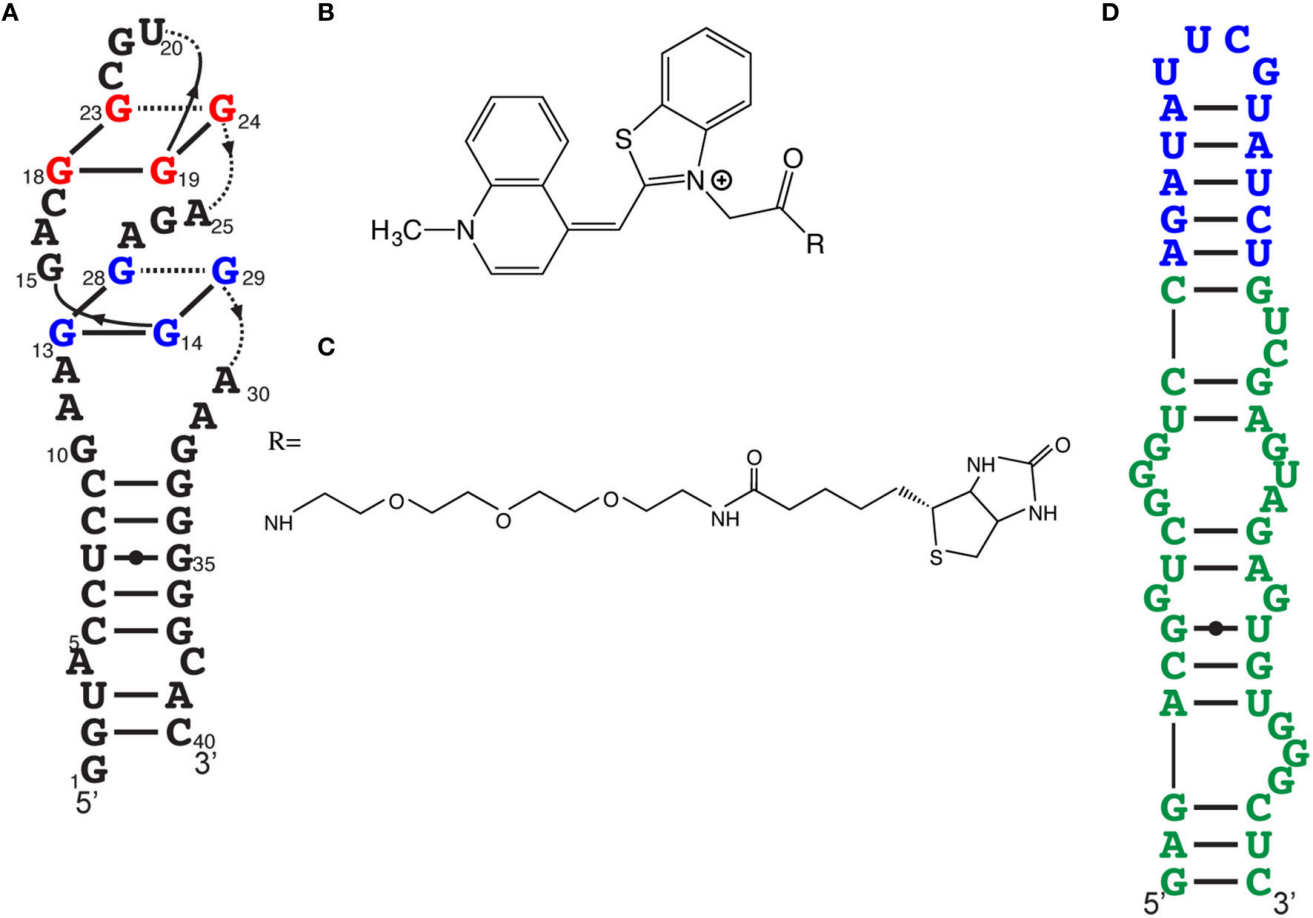
**Other efficient light-up aptamers. (A)** RNA Mango aptamer predicted secondary structure. **(B)** Acetylated thiazole orange (TO1), the fluorogen moiety of the Mango ligand. **(C)** PEGylated biotin, one of the many derivatives essayed, resulting to TO1-biotin, the ligand for RNA Mango. **(D)** Predicted secondary structure of Broccoli (in green). Effective fluorogens for Broccoli can be DFHBI or DFHBI-1T. The stem-loop in blue is the insertion point to transform this light-up aptamer as a metabolite sensor.

The fluorescence intensity of a light-up aptamer directly depends on the capacity of the aptamer to bind its cognate fluorogen. The fluorescence efficiency may therefore not only depend on brightness (the product of the molar extinction coefficient and the fluorescent quantum yield) but also on the dissociation constant (*K*_*d*_) of the fluorogen and the aptamer. Very short incubation times and stringent conditions during the selection of an aptamer against a thiazole orange derivative that was acetylated (TO1) then biotinylated (TO1-Biotin) allowed the development of the high-affinity light-up aptamer RNA Mango (Figures 4A,B,C; Dolgosheina et al., 2014). This 39 nucleotide-long RNA exhibits a pattern of four regularly spaced G pairs, suggesting a G-quadruplex. Circular dichroic spectra suggest a compact potassium-dependent parallel G-quadruplex upon TO1-Biotin binding. Injection of TO1-Biotin in *C. elegans* syncytial gonads showed bright fluorescence with RNA Mango for more than a 2-h period.

After a few “regular” rounds of SELEX, light-up aptamers were selected both for binding and fluorescence activation by using fluorescence-activated cell sorting (FACS) where the libraries were transformed in *E. coli*, with and without DFHBI (Filonov et al., 2014). This double-selection approach led to a new light-up RNA of better affinity than Spinach2 as well as a higher *T*_*m*_ (48°C instead of 37°C). This 49 nucleotide-long RNA, named Broccoli (Figure 4D), activates the fluorescence of either DFHBI or DFHBI-1T. This combined SELEX-FACS approach on both aptamer selection (binding) and screening (fluorescence activation) was rapid but, more importantly, allowed selection of RNA with improved stability in cellular environments (Bartel et al., 1991). For example, Broccoli does not require the use of a tRNA scaffold (required for Spinach imaging *in vivo*) and it has a reduced magnesium dependence, which contributes to a nearly 2-fold brightness increase in *E. coli*. Additionally, the secondary structure of Broccoli, containing a hairpin stem-loop, was engineered to form a dimer by substituting the terminal loop with a second Broccoli aptamer resulting in a 70% increase in fluorescence.

## Applications of light-up aptamers—*in vivo*

As mentioned previously, the Jaffrey laboratory not only developed Spinach, Spinach2, and Broccoli, but the research group was essential to converting the light-up aptamer into a protein-free metabolite sensor. This opened the way for sensing the abundance, distribution, and flux of intracellular molecules. Transforming Spinach-DFHBI fluorescence in an allosteric fashion, *via* the fusion of a metabolite-binding aptamer to Spinach, is a powerful application that seems to be more popular than the actual imaging of RNA trafficking *in vivo*. Light-up aptamers have been used to monitor, *in vitro*, and/or in live *E. coli*, the following metabolites: adenosine, ADP, guanine, SAM, guanine, and GTP (Paige et al., 2012); cyclic-di-GMP (Nakayama et al., 2012; Kellenberger et al., 2013) and c-AMP-GMP (Kellenberger et al., 2013).

In addition to only RNA molecules, the light-up aptamers have been used to monitor proteins: thrombin, streptavidin, and the MS2 phage coat protein (MCP; Song et al., 2013). The *in vitro* detection of protein concentrations of thrombin using the thrombin sensor (a thrombin aptamer fused with a transducer to the Spinach RNA) in solution showed affinity in the nanomolar range. For example, detection of expression of streptavidin in *E. coli* resulted in a 10-fold increase of the fluorescence signal when the streptavidin light-up aptamer was co-expressed. An immediate concern might be about the robustness of the quantitative protein concentration determination from such light-up aptamers. To alleviate uncertainty, a linear correlation between the fluorescence generated by the MCP light-up aptamer and the MCP protein levels (as measured by western blot) indicated that the MCP light-up aptamer is as reliable as direct readout of protein levels, without the need of additional antibody chemistry. The MCP sensor was able to show cell-to-cell MCP synthesis variability, indicating the use of this technology in single-cell interactions within population studies and may also provide a new approach to study molecular crowding.

Light-up aptamers have been successfully engineered to detect metabolites as well as proteins in bacteria; doing the same in mammalian cell is however much more complicated. So far, live cell imaging of several RNAs has been performed in various studies, such as with the 5S RNA (Paige et al., 2011), the 7SK that localizes to nuclear speckles as well as CGG repeat-containing RNAs leading to RNA aggregates (where disaggregation of foci is observed within 1 h of tautomycin addition; Strack et al., 2013) and the 6S transcriptional control RNA to which RNA Mango was fused into a non-conserved stem-loop structure (Dolgosheina et al., 2014).

The common feature of those aforementioned studies in mammalian cells consists of highly abundant RNAs, a marginal solution to increase the signal-to-noise ratio. In fact, Spinach-tagged RNA expressed by the CMV promoter (a Pol II promoter) was not detected in mammalian Mv1Lu cells nor in yeast (Ilgu et al., 2015). To drastically improve the signal-to-noise ratio while measuring Spinach-tagged Pol II expressed mRNAs, a different research group used a spinning-disk confocal microscopy acquisition, coupled to a space- and time- adaptive denoising algorithm (Guet et al., 2015) to successfully monitor RNA trafficking. Moreover, the cell-to-cell heterogeneity of the ligand permeability, of the transient transfection as well as of the specific cellular cycle position creates cell-to-cell variation in fluorescence background. Very few articles quantified the eukaryotic cell permeability of the fluorogens. Currently, it is known that DFHBI shows similar permeability kinetics to Hoechst in COS-7 cells, where it reached a fluorescence intensity plateau after 30 min (Strack et al., 2013). Another major bias in results interpretation may come from the background subtractions process and therefore, great care must be taken during microscopy image analysis. Raw images of entire fields (including the fluorogen without aptamer as well as the mCherry transfection controls) would alleviate interrogations about such problems and would also allow monitoring of the cell-to-cell variability of the fluorescence signal (for example, the size, number, and distribution of aggregates) and thus, helping to move the field forward. Clearly, live RNA imaging in eukaryotic cells is currently a challenge with light-up aptamers, a problem also apparent from the very few articles published on eukaryotic light-up aptamer in comparison to bacteria. This problem (mostly due to the low intensity of fluorescence in eukaryotic cells) is being addressed on parallel fronts with better microscopy techniques, better aptamers and better fluorogens.

Additional experimental strategies to detect cellular mRNA using RNA aptamers and fluorophores were developed at the same time as the emergence of the new light-up aptamers. Although they don't directly qualify as light-up aptamers, the similarities between those systems are appropriate for this review. As alluded previously, RNA polymerase II express mRNA at high- as well as low-level. In an effort to alleviate this problem, the very bright fluorophores Cy3 and Cy5 were used indirectly to bind mRNA-encoding multiple-alternating aptamers against tobramycin and PDC [2- [[(3- Aminophenyl)methyl]amino]- 6- (2,6- dichlorophenyl)- 8- methyl-pyrido[2,3-d]pyrimidin-7(8H)-one]. This system has been named IMAGEtag (Intracellular MultiAptamer GEnetic tags; Shin et al., 2014). Cy3-tobramycin and Cy5-PDC can therefore be specifically localized on the mRNA and visualized by FRET (fluorescence resonance energy transfer). Unlike light-up aptamers, this approach does not photochemically convert a non-fluorescent molecule into a fluorescent one, but optical separation of the donor and acceptor signal under a microscope allows FRET imaging (leading to a drastic background reduction). In a recent study where MG and Spinach aptamers as well as IMAGEtags were compared to one another in imaging Pol II transcribed RNAs, IMAGEtags led to a much stronger signal-to-noise ratio (Ilgu et al., 2015). Although the number of aptamers present in each system was different, MGA and Spinach lead to fluorescence intensity close to background, while the very bright Cy3 and Cy5 allowed clear and distinct FRET signals.

Another similar FRET activation approach utilizes a molecular beacon (using a fluorophore-quencher pair), where the fluorophore becomes activated when the quencher binds to an endogenously produced aptamer at the 3′-end of an RNA of interest, offers the possibility of monitoring many types of mRNA *in vivo* (Arora et al., 2015).

## Applications of light-up aptamers—*in vitro*

Although still in development, there are no doubts that developing light-up aptamers for *in vivo* purpose is much harder than *in vitro*. There are multiple reasons, such as having a controlled environment instead of a dynamic organism, having non-specific binding with cellular components, unknowingly activating pathways, variable permeability of membrane(s), etc. More specifically with light-up aptamers, the *in vitro* assays allows for excellent sensors as the background fluorescence is very low, leading to a strong signal-to-noise ratio. Moreover, those aptamers simply require *in vitro* transcription to generate the needed protein-free sensor. Several *in vitro* molecular biology applications (described below) have been developed with these aptamers, such as in-gel imaging (similar to SYBR Gold), radioactivity-free kinetic activity measurements of ribozymes, affinity purification of RNA, and ribonucleoprotein complexes, identification of the ligands from orphan riboswitches and also, development of a high-throughput *in vitro* screening to identify the new drugs binding to RNA-modifying enzymes.

DFHBI-1T (Figure 2F) has been used for simple and sensitive in-gel imaging of RNAs (Filonov et al., 2015). On a native polyacrylamide gel or even polyacrylamide gels containing 7 M urea where Broccoli and Spinach2 became fluorescent by washing the gel with water (to remove urea) and the gel being soaked in presence of DFHBI-1T and appropriate salt conditions to allow the refolding of the aptamers. The detection limit for DFHBI-1T staining is ~1 fmol, which is comparable with the sensitivity of SYBR Gold. The in-gel imaging using a ChemiDoc MP (Bio-Rad) is a cost effective alternative to conventional Northern blotting where the kit and membranes ($469 at NorthernMax Kit, Life Technologies) has a cost of $11.70 per blot, and DFHBI-1T (5 mg at $399.99 at Lucerna Technologies, New York) has a cost $0.80 per gel. This simple and cost effective gel monitoring of RNA proved to be a rapid way to investigate the stability of RNA with different RNA scaffold, resulting in the development of a modified, more versatile, and stable RNA scaffold F30 stable both in *E. coli* and mammalian cells (Filonov et al., 2015). Moreover, it allowed rapid optimization of the Broccoli aptamer to increase its brightness as well.

A technique that commonly requires gel imaging is ribozyme cleavage activity where the RNA is radiolabeled (usually with ^32^P). Modified light-up aptamers have been developed for non-isotopic detection of the hammerhead ribozyme self-cleavage in real-time by fluorescence. Extra sequences are added to the hammerhead ribozyme to bind one half of the sequence of Spinach. Once the ribozyme self-cleaves, the half-Spinach sequence is released and binds its cognate Spinach second half to reconstitute a bi-molecular Spinach aptamer to induce fluorescence activation upon DFHBI binding (Ausländer et al., 2016). The hammerhead ribozyme cleavage activity can therefore be monitored in real-time in a fluorimeter, rather than quenching radioactive aliquots that are resolved on a gel and then imaged with expensive instrumentation.

Biotin is commonly used with streptavidin for affinity purification of complexes. The Mango aptamer binds TO1-Biotin, providing a strong binding domain for purification using streptavidin beads. Monitoring of 6S:holoenzyme complex formation as well as the purification of 6S RNA from total RNA has been performed (Filonov et al., 2014). The light-up aptamer not only allowed the purification of the RNA, it also provided a visual confirmation of the purification in progress.

Many riboswitches have been proposed by bioinformatics but their cognate ligands are still unknown (Weinberg et al., 2010). Since the core of the ligand recognition and binding site of riboswitches is composed of an aptamer, identifying the ligand that binds such aptamer would complete the riboswitch identification and better understand the regulation of the downstream gene. An engineered Spinach-based riboswitch has been developed for metabolite dynamics imaging in living cells (You et al., 2015). In a traditional riboswitch approach, the presence of the riboswitch cognate metabolite would activate or inhibit the production of a protein (usually GFP) *in vivo*. Here, use of a strict protein-free system might be a powerful *in vitro* tool to identify the ligands of orphan riboswitches that have been proposed by bioinformatics.

*In vitro* transcription activity can also be monitored where the beginning of a transcript contains a misfolded Spinach followed by a hammerhead ribozyme. The ribozyme self-cleavage release the RNA segment producing a misfolded Spinach, allowing the Spinach aptamer to fold properly and induce fluorescence upon DFHBI binding (Höfer et al., 2013). The presence of RNA can also be monitored with a bi-molecular Spinach using a similar strategy where the transcript contains an inactive Spinach misfolded by extra RNA sequences. These sequences can target an RNA of interest, and the strand displacement would permit Spinach to fold and bind DFHBI (Bhadra and Ellington, 2015). This *in vitro* detection of cognate RNA using this modified Spinach aptamer was very specific as it demonstrated that it could even distinguish single-nucleotide mismatches.

A recent example illustrating how this protein-free technology might accelerate the identification of drug candidates is with a high-throughput approach. A m^6^A methylated Broccoli is non-fluorescent, but the presence of the demethylase ALKBH5 recovered the fluorescence activity of Broccoli (Svensen and Jaffrey, 2016). Being a simple experiment, this approach was converted into a high-throughput screen where small molecules were added. Several of those molecules prevented the fluorescence activation, identifying them as demethylase ALKBH5 inhibitors. The simplification of the detection offered by light-up aptamers is promising to discover new activator or inhibitors of other complex cellular regulatory proteins.

## Conclusion

Cellular imaging with GFP allowed better understanding of protein expression, interaction, localization, and role into cells as well as beyond the cell. SELEX has brought a multitude of aptamers, including aptamers that increase the fluorescence intensity of otherwise non-fluorescent molecules. Spinach2, Baby Spinach, and Broccoli activate the fluorescence of HBI derivatives (the compound naturally formed in native GFP). Moreover, Mango RNA aptamer selectively activates a thiazole orange derivative with high affinity. We are now entering in a new field of protein-free fluorescence detection of endogenously produced molecules. The *in vitro* applications are very diverse and include visually-guided affinity purification, in-gel imaging, ribozyme cleavage activity real-time measurements, screening of new RNA-modifying drugs, and riboswitches among others. The *in vitro* low background and high signal-to-noise ratio are the main factors helping to rapidly develop this field. The *in vivo* applications are still clearly separated between the prokaryotic and eukaryotic cells. Publications from various research groups undeniably confirm the robustness and the usefulness of the light-up aptamers in bacteria (mostly for metabolite sensing). It should however be noted that under certain conditions, a single Spinach fluorescence may be barely above background (Zhang et al., 2015); indicating that signal-to-noise may be problematic, even with bacteria. However, light-up aptamers in eukaryotic cells represent a clear and major challenge with an extremely low signal-to-noise ratio. Highly abundant RNA or RNA aggregates (nucleotide triplets) were successfully measured. Microscopy techniques, such as fast spinning disk confocal fluorescence followed by image processing, can alleviate some of the current hurdles, but much has to be improved to make that technique widely viable. Among other things, the development of optimized aptamers and optimized fluorogens may contribute to get better *in vivo* fluorescence signals, allowing new applications. The advent of more efficient and high-performing parallel imaging technologies, such as the one found in Illumina's NGS technology (Liu et al., 2012) will surely lead to the discovery of new light-up aptamers and fluorogens.

While the number (and quality) of applications *in vitro* and in bacteria will surely continue to increase over the next few years, several *in vivo* challenges must be prioritized for the light-up aptamers. Among others, low-level Pol II expressed mRNA must be easily monitored, transfection controls, and wide field view of raw images must be constantly be present in publication to identify the cell-to-cell fluorescence variation, which is crucial for the background subtraction. In the meantime, it is undeniably a great tool to consider for protein-free *in vitro* fluorescence applications.

## Author contributions

JO was the sole writer of this review.

### Conflict of interest statement

The author declares that the research was conducted in the absence of any commercial or financial relationships that could be construed as a potential conflict of interest.
